# Synthesis, Biological Activity, and Apoptotic Properties of NO-Donor/Enmein-Type *ent*-Kauranoid Hybrids

**DOI:** 10.3390/ijms17060747

**Published:** 2016-05-24

**Authors:** Dahong Li, Xu Hu, Tong Han, Shengtao Xu, Tingting Zhou, Zhenzhong Wang, Keguang Cheng, Zhanlin Li, Huiming Hua, Wei Xiao, Jinyi Xu

**Affiliations:** 1 Key Laboratory of Structure-Based Drug Design & Discovery, Ministry of Education, and School of Traditional Chinese Materia Medica, Shenyang Pharmaceutical University, Shenyang 110016, China; lidahong0203@163.com (D.L.); huxu105@163.com (X.H.); hantong1221@163.com (T.H.); tingtingzhou1118@hotmail.com (T.Z.); lzl1030@hotmail.com (Z.L.); 2 State Key Laboratory of New-Tech for Chinese Medicine Pharmaceutical Processes, and National Post-Doctoral Research Workstation, Jiangsu Kanion Pharmaceutical Co., Ltd., Lianyungang 222001, China; kanionxw2010@126.com; 3 State Key Laboratory for the Chemistry and Molecular Engineering of Medicinal Resources, and School of Chemistry and Pharmacy, Guangxi Normal University, Guilin 541004, China; kgcheng2008@gmail.com; 4 Department of Medicinal Chemistry and State Key Laboratory of Natural Medicines, China Pharmaceutical University, Nanjing 210009, China; cpuxst@163.com

**Keywords:** NO-donor, enmein-type, *ent*-kauranoid, antiproliferative, apoptosis

## Abstract

Herein, we reported on a series of synthetic nitric oxide-releasing enmein-type diterpenoid hybrids (**9a**–**i**). All the target compounds showed potent antibacterial activity against selected Gram-positive bacteria *S. aureus* and *B. subtilis*. The antiproliferative activity against human tumor K562, MGC-803, CaEs-17 and Bel-7402 cells, and human normal liver cells L-02 was tested and the structure activity relationships (SARs) were also concluded. Compounds **9b** and **9d** showed the best activity against *S. aureus* and *B. subtilis* with the same minimal inhibitory concentrations (MICs) of 4 and 2 μg/mL, respectively. The derivative **9f** displayed IC_50_ values of 1.68, 1.11, 3.60 and 0.72 μM against the four cancer cell lines above and 18.80 μM against normal liver cells L-02; meanwhile, **9f** also released a high level of NO at the time point of 60 min of 22.24 μmol/L. Furthermore, it was also found that **9f** induced apoptosis *via* the mitochondria-related pathway and arrested cell cycle of Bel-7402 cells at S phase. These findings might be important to explore new chemical entities for the main causes of in-hospital mortality of *S. aureus* infection, combined with a solid tumor.

## 1. Introduction

Bacterial infections, causing deadly diseases, have increased at an alarming rate [1,2]. The development of drug resistance among the infectious bacterial strains and absence of effective preventive measures have become the two major driving forces for design and identify suitable active new chemical entities. On the other hand, cancer, uncontrolled, rapidly proliferating, abnormal cells, is now the second leading cause of human deaths, and the high mortality rate engages with the rising number of diverse cancer types [3]. These have triggered research aiming to find new lead structures that may be beneficial in the design of novel antitumor agents [4,5]. Patients with neoplastic disorders are subjected to chemotherapeutic treatment, and susceptible to microbial infections due to the poor immunity [6]. For example, *Staphylococcus aureus* is a pathogen responsible for the main causes of in-hospital mortality [7]. Therefore, the discovery of new novel potent antibacterial and antitumor chemical entities is challenging work for medicinal chemists [8,9,10,11].

A potential solution is to explore innovative natural scaffolds from natural sources [12,13]. Among numerous exploited medicinal plants, the *Isodon* species are traditionally used as antitumor and antibacterial folk medicine and have many complex and bioactive diterpenoids [14,15]. They have recently received great attention from researchers from many research fields [16,17,18]. In this context, enmein-type *ent*-kauranoids, one of the important and main classes of diterpenoids from the plants of *Isodon* species with antitumor and antibacterial activities, are worthy of intensive investigation.

Nitric oxide (NO) is an endogenous, small and reactive molecule, which has various physiological and biological properties [19,20,21]. High concentration of NO is cytotoxic to tumor cells by inducing tumor cell apoptosis, sensitizing drug-resistant tumor cells, and inhibiting tumor metastasis, and so on [22,23]. NO also exhibits antimicrobial activity and kills bacteria through lipid peroxidation, DNA cleavage, and protein dysfunction. The multiple bactericidal pathways of NO make it a potent broad-spectrum antimicrobial agent with low risk [24,25,26]. Thus, NO-donating groups are good pharmacophores for both antitumor and antibacterial agents.

On the basis of the above, we developed a series of new enmein-type *ent*-kauranoid derivatives containing furozan-based NO-donors. The antibacterial potency, antiproliferative activity and NO releasing ability of target compounds were tested. Furthermore, the preliminary antitumor mechanisms were also disclosed.

## 2. Results and Discussion

### 2.1. Chemistry

**Scheme 1:**
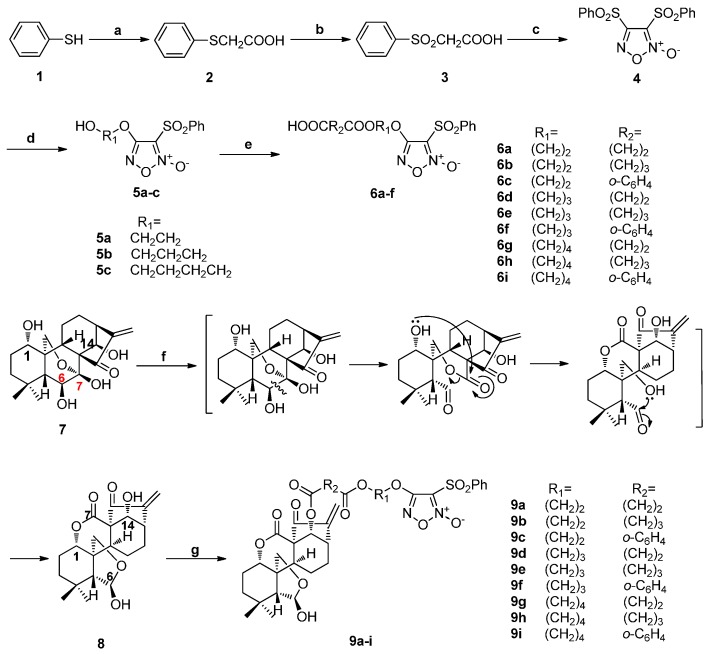
Synthesis of NO donor/enmein-type *ent*-kauranoid hybrids **9a**–**i**. Reagents and conditions: (**a**) ClCH_2_COOH, NaOH(aq), 140 °C, 2 h; (**b**) 30% H_2_O_2_, AcOH, rt, 3 h; (**c**) fuming HNO_3_, 90 °C, 4 h; (**d**) diol, THF, 30% NaOH, 0 °C, 4–8 h; (**e**) triethylamine, succinic anhydride, DMAP, rt, 1 h; (**f**) NaIO_4_, H_2_O, rt, 6 h; (**g**) **6**, EDCI, DMAP, rt, 12 h.

Diphenylsulfonylfuroxan (**4**) was obtained in a three-step sequence starting from benzenethiol (**1**), and was then treated with corresponding diol to afford monophenylsulfonylfuroxans (**5a**–**c**). Furoxan-based NO donors intermediate **6a**–**i** were obtained from the condensation of **5a**–**c** with corresponding anhydride. Enmein-type *ent*-kauranoid derivative **8** was synthesized from oridonin (**7**) by treatment with sodium periodate in water. Target NO donor/enmein-type *ent*-kauranoid hybrids **9a**–**i** were designed and synthesized from **8** with **6a**–**i** in the presence of DMAP/EDCI in DCM. Flash chromatography was used only at the last step of the synthetic route of each target compound (Scheme 1) [22,27].

### 2.2. Antimicrobial Activity

The antibacterial activity of NO donor/enmein-type *ent*-kauranoid hybrids **9a**–**i** against the Gram-negative bacterium *Escherichia coli* (ATCC 25922), Gram-positive bacterium *Staphylococcus aureus* (ATCC 29213) and *Bacillus subtilis* (CMCC 63501), and the fungus *Monilia albicans* (ATCC 10231) was first disclosed and summarized in Table 1. Parent compound enmein-type 6,7-*seco*-*ent*-kauranoid **8** showed the minimum inhibitory concentration (MIC) above 100 μg/mL and derivatives **9a**–**i** exhibited antibacterial activity against gram-positive bacterium *S. aureus* and *B. subtilis* to some extent. In the *S. aureus* strain, **9a**–**i** exhibited similar or stronger antibacterial activity than **7**, which meant that structural modifications introduced going in the right direction, improving its biological properties. In the *B. subtilis* strain, **9b**–**d** and **9i** were even more potent than positive control chloromycetin. Thus, the introduction of NO-donor substituent groups at the 14-position of enmein-type 6,7-*seco*-*ent*-kauranoid could improve antimicrobial activity against *S. aureus* and *B. subtilis*. However, almost all of them were inactive (MIC > 100 μg/mL) against *E. coli* and *M. albicans*, and target compounds selectively inhibited Gram-positive bacteria *S. aureus* and *B. Subtilis*.

The most promising compounds **9b** and **9d** exhibited the strongest antibacterial activity among **9a**–**i** with MICs of 4 and 2 μg/mL against *S. aureus* and *B. subtilis*. The substituents (R_1_ and R_2_) of **9b** were (CH_2_)_2_ and (CH_2_)_3_, respectively, while the substituents were (CH_2_)_3_ and (CH_2_)_2_ of **9d**. Coincidentally, two of the most active compounds, **9b** and **9d**, had the same total linkage length of five carbons (CH_2_)_5_. The linkages between NO donor and parent compound always affected the biological activity. Different lead compounds had their own favorable linker [22,23,28,29].

### 2.3. Antiproliferative Activity

The antiproliferative activity of **9a**–**i** against four human cancer cell lines K562 leukemia cell line, MGC-803 gastric cancer cell line, CaEs-17 esophageal cancer cell line, and Bel-7402 hepatoma cell line, and human normal liver cells L-02 was evaluated by MTT assay (Table 2). All the target molecules **9a**–**i** were stronger than oridonin **7** with IC_50_ values below 1.33 μM and lower than Taxol (1.89 μM) against Bel-7402 cell line. The most potent **9f** with IC_50_ of 0.72 μM was 1-fold stronger than Taxol. While, target compounds were not so sensitive to CaEs-17 cells with IC_50_ ranging from 3.60 to 5.13 μM. IC_50_ values against MGC-803 cell line were a little weaker than Taxol, between 1.11 to 1.83 μM. For K562 cell line, the antiproliferative activity of all the derivatives was weaker than Taxol. Most NO donor derivatives showed cytotoxic selectivity between tumor and normal liver cells to some extent, with IC_50_ values ranging from 10.36 to 19.87 μM against L-02 normal liver cells. These results confirmed previous reports [28,29] that some NO donating derivatives showed less cytotoxicity to normal cells.

In most cases against the selected tumor cell lines, among the derivatives **9a**–**i**, when the substitution R_1_ was the same (**9a**–**c**, **9d**–**f** and **9g**–**i**) and R_2_ was changed among (CH_2_)_2_, (CH_2_)_3_, and *o*-C_6_H_4_ in each group, compounds (**9c**, **9f** and **9i**) with R_2_ of aromatic group of *o*-C_6_H_4_ showed stronger activity than corresponding ones with alkyl groups (**9a**, **9b**, **9d**, **9e**, **9g** and **9h**). Between alkyl substituents (CH_2_)_2_ and (CH_2_)_3_ in R_2_, the latter one (**9b**, **9e** and **9h**) was favorable. For example, **9i** with the same R_1_ of (CH_2_)_4_ as **9g** and **9h**, and R_2_ of *o*-C_6_H_4_, showed slightly lower IC_50_ values of 1.25, 1.86, 3.82 and 0.78 μM against K562, MGC-803, CaEs-17 and Bel-7402 tumor cells, respectively. When the substitution R_2_ was the same and R_1_ was changed among (CH_2_)_2_, (CH_2_)_3_, and (CH_2_)_4_, compounds showed statistically equal IC_50_ values.

### 2.4. NO-Releasing Ability

**Figure 1:**
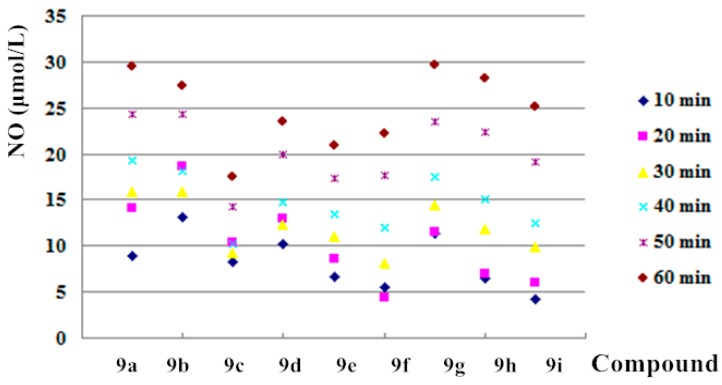
NO-releasing ability of compounds **9a**–**i**.

The NO-releasing ability of **9a**–**i** was determined by Griess assay at 100 μM and measured at the time point of 10, 20, 30, 40, 50 and 60 min (Figure 1). The concentrations of released NO for all synthetic hybrids increased with time and were more than 20 μmol/L (except **9c**) at the time point of 60 min. High NO releasing ability might, at least to a certain extent, contribute to antiproliferative and antibacterial activities [23,26]. The most promising NO-releasing enmein-type 6,7-*seco*-*ent*-kauranoid derivative was **9f** with the highest selectivity index (SI) value of 26.1. It was chosen for intensive mechanism study on hepatoma Bel-7402 cell line.

### 2.5. Influence of **9f** on the Bel-7402 Cell Cycle

**Figure 2:**
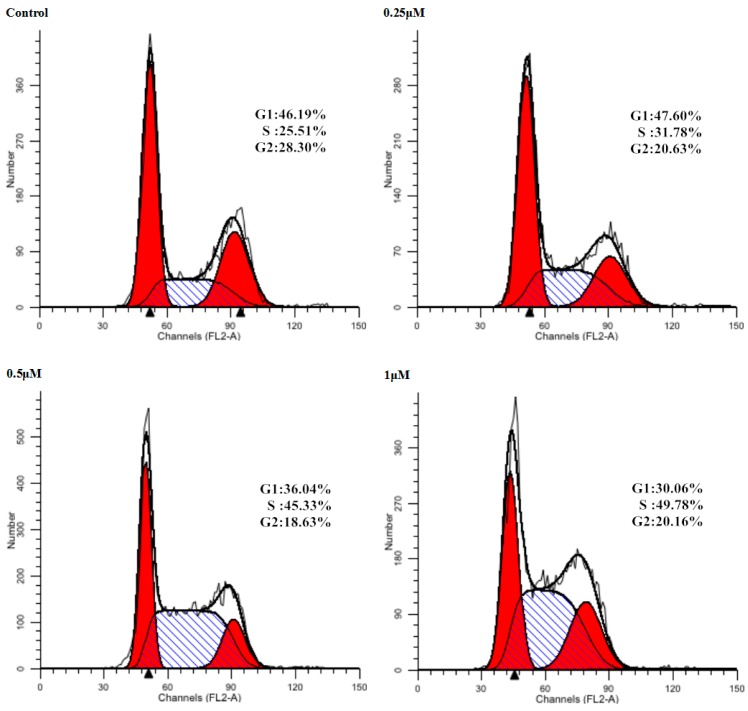
Influence of Bel-7402 cell cycle by **9f**. Left red part: cells in G_1_ phase; Right red part: cells in G_2_ phase; Oblique line part: cells in S phase; White part: total cells.

Cell cycle arrest is an important sign for inhibition of proliferation and the series of events that take place in a cell leading to its division and replication. Some NO donating hybrids exhibited cell cycle arrest properties [28]. As the inhibitory effect of **9f** on cell proliferation was observed, we next assessed the effect on the cell cycle distribution of Bel-7402 cells by flow cytometry (Figure 2). Treatment of Bel-7402 cells with **9f** at 0.25, 0.5 and 1 μM resulted in a remarkable increase in the percentage of cells in S/G_2_ phase from 25.51% of control group to 31.78%, 45.33%, and 49.78%, respectively. Compound **9f** could influence Bel-7402 cell cycle progression at low micromolar concentrations in a dose-dependent manner.

### 2.6. Induction of Apoptosis by **9f**

**Figure 3:**
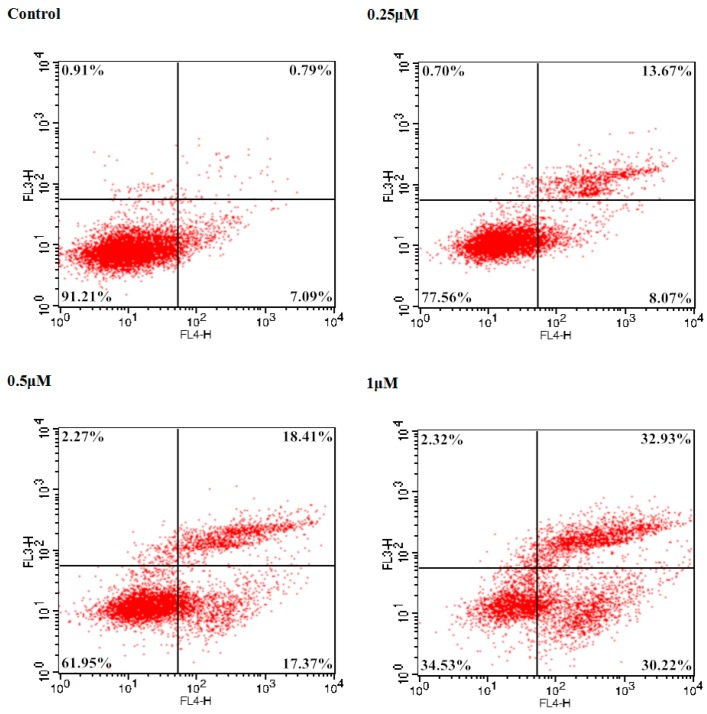
Induction of apoptosis by **9f** in Bel-7402 cells.

To examine the potent cancer cell antiproliferative effect of **9f** on Bel-7402 cells, the number of apoptotic cells was monitored using flow cytometry. The total percentage of apoptotic cells (early and late, Q2 + Q4) was 7.88% treated with a vehicle alone. In comparison with the control group, 2.76-, 4.54-, and 8.01-fold percentages of apoptotic cells were observed when different concentrations (0.25, 0.5 and 1 μM) of **9f** were added to Bel-7402 cells. As shown in Figure 3, compound **9f** caused significant induction of apoptosis in a dose-dependent manner in Bel-7402 cells and resulted in 21.74%, 35.78% and 63.15% apoptotic cells, respectively.

### 2.7. **9f** Induced Mitochondrial Depolarization

**Figure 4:**
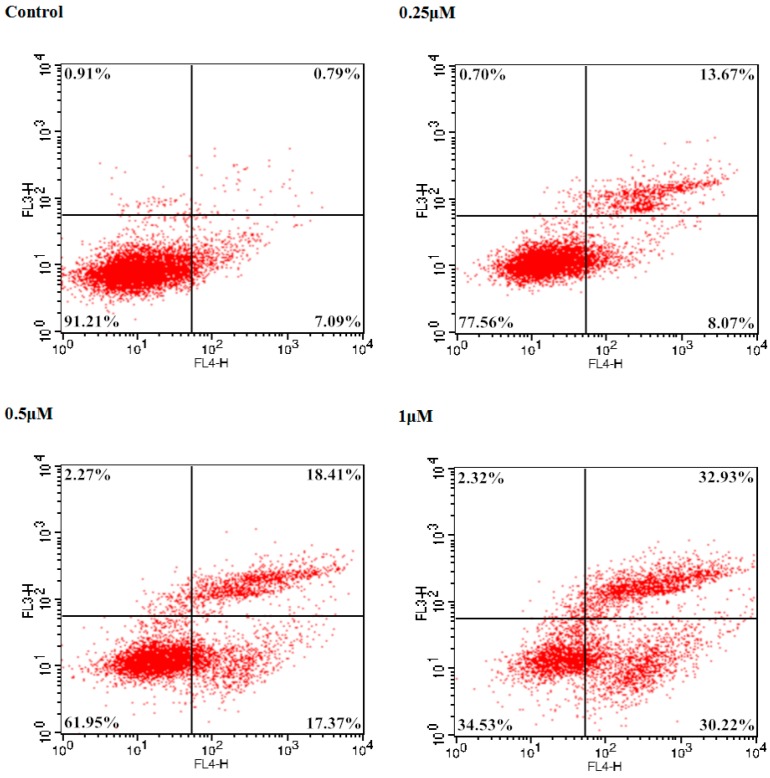
**9f** induced mitochondrial depolarization in Bel-7402 cells.

In the field of cancer, cellular apoptosis plays a very important role. The loss of mitochondrial membrane potential is a key event during drug-induced apoptosis. In order to investigate the mitochondria related effect of **9f**, the fluorescent probe JC-1 (Keygen, KGA601, Nanjing, China) was used to detect the changes on mitochondrial membrane potential. Before Bel-7402 cells were stained with JC-1, cells were incubated with 0, 0.15, 0.3, and 0.6 μM of **9f**. Then, the flow cytometry analysis was carried out to determine the cell numbers with collapsed mitochondria in different cell groups (Figure 4). The percentage of apoptotic cells increased in a dose-dependent fashion and showed 4.62%, 8.81%, 21.82% and 25.98%, respectively. These results together with Annexin-V and PI double stain apoptosis assay confirmed that **9f** could induce apoptosis in a Bel-7402 cells mitochondria-related pathway.

## 3. Materials and Methods

### 3.1. Chemistry

#### 3.1.1. General

All commercially available solvents and reagents were purchased from a local commercial supplier (Yuwang Chemical Industries, Ltd., Shenyang, China) and used directly. Melting points (m.p.) were tested using micro melting point apparatus XT-4 (Taike, Beijing, China) and not corrected. Infrared (IR) spectra were recorded on a Nicolet Impact 410 instrument (Thermo Fisher Scientific, Waltham, MA, USA) using a KBr pellet. ^1^H and ^13^C NMR spectra were taken (TMS as internal standard) on a Bruker AV-300 (Bruker Corp., Karlsruhe, Germany) or ACF 500 spectrometer (Bruker Corp., Karlsruhe, Germany) in CDCl_3_. Low resolution mass spectra (MS) were carried out using FTMS-2000 (Thermo Fisher Scientific, Waltham, MA, USA). High resolution mass spectra (HR-MS) were obtained with Agilent QTOF 6520 (Agilent Technologies China, Beijing, China).

#### 3.1.2. General Procedure to Synthesize **9a**–**i**

Compound **8** (72 mg, 0.2 mmol) was dissolved in 15 mL of dichloromethane and mixed with **6a**–**i** (0.24 mmol), EDCI and DMAP, stirring at room temperature for 8 h. When the reaction was finished (monitored by TLC), the reaction mixture was poured into 10% HCl (about 15 mL), and then extracted three times (each 10 mL) with dichloromethane. The organic phases were combined together, washed with water (about 15 mL) and saturated brine (about 15 mL), sequentially, then dried over anhydrous Na_2_SO_4_, and evaporated to dryness in reduced pressure. At the last step, the product was purified by flash column chromatography (CH_2_Cl_2_/MeOH *v*/*v* 300:1) to obtain the target molecules.

Compound **9a**: white solid, 48% yield: m.p. 92 °C–94 °C; IR (KBr) υ_max_ 3439, 2959, 2025, 1723, 1615, 1553, 1450, 1371, 734, 685 cm^−1^; ^13^C NMR (CDCl_3_, 100 MHz), δ (ppm) 198.07, 172.91, 172.69, 167.22, 158.99, 147.86, 138.18, 135.83, 129.89 (×2), 128.68 (×2), 120.53, 110.61, 101.90, 76.18, 74.67, 74.09, 68.13, 61.46, 60.34, 53.93, 49.96, 48.60, 40.77, 37.16, 33.12, 33.03, 31.72, 31.13, 29.89, 23.33, 23.16, 19.74; ^1^H NMR (CDCl_3_, 300 MHz), δ (ppm) 8.07 (2H, d, *J* = 7.5 Hz, 2′,6′-Ar–H), 7.78 (1H, d, *J* = 7.5 Hz, 4′-Ar–H), 7.64 (2H, t, *J* = 7.5 Hz, 3′,5′-Ar–H), 6.20 (1H, s, 6-OH), 5.74 (1H, s, 17-CH_2_), 5.62 (1H, s, 17-CH_2_), 5.32 (1H, s, 6-CH), 4.47~4.65 (6H, m, overlapped, 1-CH, 14-CH, 25,26-CH_2_), 4.05, 3.92 (each 1H, dd, *J*_A_ = *J*_B_ = 9.6 Hz, 20-CH_2_), 3.16 (1H, d, *J* = 9.3 Hz, 13-CH), 2.53~2.90 (4H, m, 22,23-CH_2_), 2.17 (2H, m, 12-CH_2_), 1.97 (1H, m, 5-CH), 1.61~1.74 (5H, m, 3,11-CH_2_, 9-CH), 1.45 (2H, m, 2-CH_2_), 1.01 (3H, s, 18-CH_3_), 0.95 (3H, s, 19-CH_3_); MS (ESI) *m*/*z*: 748.1 [M + NH_4_]^+^, 731.0 [M + H]^+^, 765.3 [M + Cl]^−^; HR-MS (ESI, M + NH_4_) *m*/*z*: calcd for C_34_H_42_N_3_O_14_S: 748.2382, found: 748.2388.

Compound **9b**: white solid, 46% yield: m.p. 88 °C–89 °C; IR (KBr) υ_max_ 3442, 2954, 2025,1746, 1618, 1553, 1452, 1358, 732, 685 cm^−1^; ^13^C NMR (CDCl_3_, 100 MHz), δ (ppm) 198.14, 172.73, 172.65, 167.27, 158.79, 147.85, 138.12, 135.87, 129.87 (×2), 128.71 (×2), 120.54, 110.52, 101.87, 76.17, 74.59, 74.12, 69.04, 61.23, 60.35, 53.90, 49.93, 48.58, 40.73, 37.12, 33.00 (×2), 32.89, 31.10, 29.85, 23.29, 23.13, 19.87, 19.61; ^1^H NMR (CDCl_3_, 500 M Hz), δ (ppm) 8.00 (2H, d, *J* = 7.5 Hz, 2′,6′-Ar–H), 7.89 (1H, d, *J* = 7.5 Hz, 4′-Ar–H), 7.74 (2H, t, *J* = 7.5 Hz, 3′,5′-Ar–H), 6.18 (1H, s, 6-OH), 5.73 (1H, s, 17-CH_2_), 5.55 (1H, s, 17-CH_2_), 5.31 (1H, s, 6-CH), 4.48~4.63 (6H, m, 1-CH, 14-CH, 26,27-CH_2_), 4.05, 3.92 (each 1H, dd, *J*_A_ = *J*_B_ = 9.0 Hz, 20-CH_2_), 3.14 (1H, d, *J* = 9.0 Hz, 13-CH), 2.38~2.73 (6H, m, overlapped, 22,23,24-CH_2_), 2.00 (2H, m, 12-CH_2_), 1.95 (1H, m, 5-CH), 1.61~1.74 (5H, m, 3,11-CH_2_, 9-CH), 1.45 (2H, m, 2-CH_2_), 1.02 (3H, s, 18-CH_3_), 0.95 (3H, s, 19-CH_3_); MS (ESI) *m*/*z*: 762.3 [M + NH_4_]^+^, 745.2 [M + H]^+^, 779.4 [M + Cl]^−^; HR-MS (ESI, M + NH_4_) *m*/*z*: calcd for C_35_H_44_N_3_O_14_S: 762.2539, found: 762.2532.

Compound **9c**: white solid, 45% yield: m.p. 137 °C–139 °C; IR (KBr) υ_max_ 3443, 2954, 2025, 1757, 1727, 1617, 1553, 1450, 735, 685 cm^−1^; ^13^C NMR (CDCl_3_, 100 MHz), δ (ppm) 198.21, 167.15, 167.06, 166.58, 158.87, 147.65, 137.95, 135.74, 131.89, 131.71, 131.19, 130.92, 130.14, 129.76 (×2), 128.87, 128.55 (×2); 120.72, 110.49, 101.80, 76.24, 75.27, 74.58, 68.88, 62.45, 60.21, 53.87, 50.14, 48.76, 40.74, 37.00, 32.93, 30.97, 31.08, 29.86, 23.29, 23.07, 19.76; ^1^H NMR (CDCl_3_, 300 M Hz), δ (ppm) 8.00 (2H, d, *J* = 7.5 Hz, 2′,6′-Ar–H), 7.81 (1H, t, *J* = 4.5 Hz, 4′-Ar–H), 7.69 (1H, t, *J* = 4.5 Hz, 22-Ar–H), 7.56 (3H, m, 21,23,24-Ar–H), 7.41 (2H, t, *J* = 7.5 Hz, 3′,5′-Ar–H), 6.24 (1H, s, 6-OH), 5.87 (1H, s, 17-CH_2_), 5.52 (1H, s, 17-CH_2_), 5.33 (1H, s, 6-CH), 4.40~4.76 (6H, m, 1-CH, 14-CH, 29,30-CH_2_), 4.07, 3.96 (each 1H, dd, *J*_A_ = *J*_B_ = 9.3 Hz, 20-CH_2_), 3.29 (1H, d, *J* = 9.6 Hz, 13-CH), 2.17 (2H, m, 12-CH_2_), 1.97 (1H, m, 5-CH), 1.52~1.80 (5H, m, 3,11-CH_2_, 9-CH), 1.41 (2H, m, 2-CH_2_), 1.02 (3H, s, 18-CH_3_), 0.98 (3H, s, 19-CH_3_); MS (ESI) *m*/*z*: 801.3 [M + Na]^+^; HR-MS (ESI, M + NH_4_) *m*/*z*: calcd for C_38_H_42_N_3_O_14_S: 796.2382, found: 796.2376.

Compound **9d**: white solid, 44% yield: m.p. 110 °C–112 °C; IR (KBr) υ_max_ 3459, 2955, 2025, 1754, 1615, 1554, 1451, 1355, 732, 685 cm^−1^; ^13^C NMR (CDCl_3_, 100 MHz), δ (ppm) 197.92, 172.08, 171.73, 167.13, 158.90, 147.69, 138.01, 135.67, 129.73 (×2), 128.60 (×2); 120.46, 110.56, 101.76, 76.04, 74.52, 74.18, 67.91, 60.35, 60.16, 53.79, 49.79, 48.47, 40.59, 36.98, 32.88, 30.99, 29.74, 29.05, 28.65, 27.81, 23.18, 23.03, 19.70; ^1^H NMR (CDCl_3_, 300 M Hz), δ (ppm) 8.03 (2H, d, *J* = 9.0 Hz, 2′,6′-Ar–H), 7.91 (1H, t, *J* = 8.1 Hz, 4′-Ar–H), 7.75 (2H, t, *J* = 9.0 Hz, 3′,5′-Ar–H), 6.04 (1H, s, 6-OH), 5.74 (1H, s, 17-CH_2_), 5.69 (1H, s, 17-CH_2_), 5.19 (1H, s, 6-CH), 4.68 (2H, m, 1,14-CH), 4.45 (2H, t, *J* = 6.0 Hz, 25-CH_2_), 4.14 (2H, t, *J* = 6.0 Hz, 27-CH_2_), 3.90, 3.54 (each 1H, dd, *J*_A_ = *J*_B_ = 9.0 Hz, 20-CH_2_), 3.14 (1H, d, *J* = 5.4 Hz, 13-CH), 2.36~2.63 (6H, m, 22,23,26-CH_2_), 2.08 (2H, m, 12-CH_2_), 1.38~1.76 (6H, m, 3,11-CH_2_, 5,9-CH), 1.43 (2H, m, 2-CH_2_), 0.93 (3H, s, 18-CH_3_), 0.85 (3H, s, 19-CH_3_); MS (ESI) *m*/*z*: 767.3 [M + Na]^+^; HR-MS (ESI, M + NH_4_) *m*/*z*: calcd for C_35_H_44_N_3_O_14_S: 762.2539, found: 762.2551.

Compound **9e**: white solid, 42% yield: m.p. 82 °C–84 °C; IR (KBr) υ_max_ 3458, 2955, 2025, 1738, 1615, 1554, 1451, 1358, 732, 685 cm^−1^; ^13^C NMR (CDCl_3_, 100 MHz), δ (ppm) 197.96, 172.78, 172.56, 167.11, 158.85, 147.73, 138.02, 135.71, 129.76 (×2), 128.53 (×2), 120.40, 110.47, 101.75, 76.05, 74.49, 73.96, 68.00, 61.33, 60.16, 53.79, 49.82, 48.46, 40.63, 37.01, 32.97, 32.89, 31.57, 30.99, 29.74, 27.88, 23.19, 23.03, 19.75, 19.60; ^1^H NMR (CDCl_3_, 300 M Hz), δ (ppm) 8.02 (2H, d, *J* = 8.4 Hz, 2′,6′-Ar–H), 7.90 (1H, t, *J* = 7.5 Hz, 4′-Ar–H), 7.74 (2H, t, *J* = 8.4 Hz, 3′,5′-Ar–H), 6.25 (1H, d, *J* = 2.2 Hz, 6-OH), 6.01 (1H, s, 17-CH_2_), 5.77 (1H, s, 14-CH), 5.68 (1H, s, 17-CH_2_), 5.19 (1H, d, *J* = 2.4 Hz, 6-CH), 4.71 (1H, m, 1-CH), 4.55 (2H, t, *J* = 6.0 Hz, 26-CH_2_), 4.12 (2H, t, *J* = 6.0 Hz, 28-CH_2_), 3.88, 3.35 (each 1H, dd, *J*_A_ = *J*_B_ = 9.0 Hz, 20-CH_2_), 3.17 (1H, d, *J* = 9.3 Hz, 13-CH), 2.21~2.51 (8H, m, overlapped, 22,23,24,27-CH_2_), 2.07 (2H, m, 12-CH_2_), 1.67~1.78 (6H, m, overlapped, 3,11-CH_2_, 5,9-CH), 1.24 (2H, m, 2-CH_2_), 0.94 (3H, s, 18-CH_3_), 0.88 (3H, s, 19-CH_3_); MS (ESI) *m*/*z*: 781.4 [M + Na]^+^; HR-MS (ESI, M + NH_4_) *m*/*z*: calcd for C_36_H_46_N_3_O_14_S: 776.2695, found: 776.2687.

Compound **9f**: white solid, 28% yield: m.p. 128 °C–130 °C; IR (KBr) υ_max_ 3445, 2956, 2025, 1758, 1723, 1616, 1554, 1451, 735, 685 cm^−1^; ^13^C NMR (CDCl_3_, 100 MHz), δ (ppm) 198.23, 167.13, 167.02, 166.55, 158.86, 147.67, 137.94, 135.77, 131.91, 131.67, 131.18, 130.94, 130.15, 129.76 (×2), 128.85, 128.54 (×2); 120.75, 110.51, 101.84, 76.27, 75.29, 74.54, 68.91, 62.48, 60.23, 53.91, 50.09, 48.78, 40.72, 37.02, 32.97, 30.99, 31.07, 29.86, 29.79, 23.30, 23.12, 19.75; ^1^H NMR (CDCl_3_, 300 M Hz), δ (ppm) 8.07 (2H, d, *J* = 7.0 Hz, 2′,6′-Ar–H), 7.75 (2H, m, 22,4′-Ar–H), 7.64 (3H, m, 21,23,24-Ar–H), 7.57 (2H, m, 3′,5′-Ar–H), 6.21 (1H, s, 6-OH), 5.92 (1H, s, 17-CH_2_), 5.56 (1H, s, 17-CH_2_), 5.32 (1H, s, 6-CH), 4.31~4.60 (6H, m, overlapped, 1-CH, 14-CH, 29,31-CH_2_), 4.08, 3.97 (each 1H, dd, *J*_A_ = *J*_B_ = 9.0 Hz, 20-CH_2_), 3.26 (1H, d, *J* = 8.7 Hz, 13-CH), 2.57~2.78 (2H, m, 30-CH_2_), 2.35 (2H, m, 12-CH_2_), 1.95 (1H, m, 5-CH), 1.42~1.78 (5H, m, 3,11-CH_2_, 9-CH), 1.24 (2H, m, 2-CH_2_), 1.03 (3H, s, 18-CH_3_), 0.96 (3H, s, 19-CH_3_); MS (ESI) *m*/*z*: 815.4 [M + Na]^+^; HR-MS (ESI, M + NH_4_) *m*/*z*: calcd for C_39_H_44_N_3_O_14_S: 810.2539, found: 810.2530.

Compound **9g**: white solid, 37% yield: m.p. 85 °C–87 °C; IR (KBr) υ_max_ 3442, 2956, 2025, 1755, 1616, 1554, 1452, 1360, 733, 685 cm^−1^; ^13^C NMR (CDCl_3_, 100 MHz), δ (ppm) 197.92, 172.21, 172.00, 167.09, 158.93, 147.69, 138.01, 135.67, 129.72 (×2), 128.56 (×2); 120.45, 110.47, 101.76, 76.03, 74.55, 74.16, 70.98, 63.86, 60.14, 53.78, 49.80, 48.46, 40.59, 36.99, 32.88, 30.99, 29.74, 29.09, 28.74, 25.18, 24.95, 23.19, 23.02, 19.73; ^1^H NMR (CDCl_3_, 300 M Hz), δ (ppm) 8.06 (2H, d, *J* = 7.8 Hz, 2′,6′-Ar–H), 7.90 (1H, t, *J* = 7.2 Hz, 4′-Ar–H), 7.65 (2H, t, *J* = 7.8 Hz, 3′,5′-Ar–H), 6.25 (1H, s, 6-OH), 6.03 (1H, s, 17-CH_2_), 5.77 (1H, s, 14-CH), 5.68 (1H, s, 17-CH_2_), 5.19 (1H, s, 6-CH), 4.62 (1H, m, 1-CH), 4.42 (2H, m, 25-CH_2_), 4.07 (2H, m, 28-CH_2_), 3.90, 3.54 (each 1H, dd, *J*_A_ = *J*_B_ = 10.5 Hz, 20-CH_2_), 3.14 (1H, d, *J* = 9.6 Hz, 13-CH), 2.47~2.54 (8H, m, overlapped, 22,23,26,27-CH_2_), 1.66~1.81 (8H, m, overlapped, 3,11,12-CH_2_, 5,9-CH), 1.24 (2H, m, 2-CH_2_), 0.93 (3H, s, 18-CH_3_), 0.88 (3H, s, 19-CH_3_); MS (ESI) *m*/*z*: 776.3 [M + NH_4_]^+^, 793.2 [M + Cl]^−^; HR-MS (ESI, M + NH_4_) *m*/*z*: calcd for C_36_H_46_N_3_O_14_S: 776.2695, found: 776.2693.

Compound **9h**: white solid, 42% yield: m.p. 78 °C–80 °C; IR (KBr) υ_max_ 3444, 2960, 2025, 1755, 1616, 1554, 1452, 1365, 732, 685 cm^−1^; ^13^C NMR (CDCl_3_, 100 MHz), δ (ppm) 198.10, 172.34, 172.11, 167.26, 159.04, 147.69, 138.16, 135.81, 129.85 (×2), 128.66 (×2); 120.58, 110.58, 101.85, 76.18, 74.57, 74.30, 71.11, 63.98, 60.28, 53.90, 49.92, 48.57, 40.71, 37.09, 32.98, 31.09, 29.84, 29.20, 28.86, 25.29, 25.05, 23.30, 23.13, 22.76, 19.83; ^1^H NMR (CDCl_3_, 300 M Hz), δ (ppm) 8.06 (2H, d, *J* = 7.2 Hz, 2′,6′-Ar–H), 7.77 (1H, t, *J* = 7.2 Hz, 4′-Ar–H), 7.63 (2H, t, *J* = 7.2 Hz, 3′,5′-Ar–H), 6.21 (1H, s, 6-OH), 5.73 (1H, s, 17-CH_2_), 5.57 (1H, s, 17-CH_2_), 5.32 (1H, m, 6-CH), 4.57 (2H, m, 1,14-CH), 4.45 (2H, t, *J* = 6.3 Hz, 26-CH_2_), 4.14 (2H, t, *J* = 6.0 Hz, 29-CH_2_), 4.06, 3.93 (each 1H, dd, *J*_A_ = *J*_B_ = 9.3 Hz, 20-CH_2_), 3.13 (1H, d, *J* = 9.6 Hz, 13-CH), 2.45~2.50 (10H, m, overlapped, 22,23,24,27,28-CH_2_), 2.35 (2H, m, 12-CH_2_), 1.58~1.98 (6H, m, overlapped, 3,11-CH_2_, 5,9-CH), 1.24 (2H, m, 2-CH_2_), 0.93 (3H, s, 18-CH_3_), 0.87 (3H, s, 19-CH_3_); MS (ESI) *m*/*z*: 795.4 [M + Na]^+^; HR-MS (ESI, M + NH_4_) *m*/*z*: calcd for C_37_H_48_N_3_O_14_S: 790.2852, found: 790.2841.

Compound **9i**: white solid, 43% yield: m.p. 126 °C–128 °C; IR (KBr) υ_max_ 3443, 2955, 2025, 1758, 1723, 1615, 1553, 1451, 734, 685 cm^−1^; ^13^C NMR (CDCl_3_, 100 MHz), δ (ppm) 198.12, 167.09, 166.90, 166.51, 158.96, 147.64, 137.97, 135.72, 131.71, 131.41, 131.22, 130.89, 130.16, 129.75 (×2), 128.64, 128.53 (×2); 120.58, 110.49, 101.76, 76.13, 74.98, 74.56, 70.94, 64.72, 60.15, 53.79, 49.97, 48.69, 40.61, 36.97, 32.88, 31.00, 29.80, 25.26, 24.95, 23.23, 23.02, 19.76; ^1^H NMR (CDCl_3_, 300 M Hz), δ (ppm) 8.07 (2H, d, *J* = 7.8 Hz, 2′,6′-Ar–H), 7.73 (3H, m, 23,25,26-Ar–H), 7.60 (2H, m, 24,4′-Ar–H), 7.50 (2H, t, *J* = 7.8 Hz, 3′,5′-Ar–H), 6.21 (1H, s, 6-OH), 5.93 (1H, s, 17-CH_2_), 5.56 (1H, s, 17-CH_2_), 5.34 (1H, m, 6-CH), 4.68 (2H, m, 1,14-CH), 4.40~4.62 (4H, m, 29,32-CH_2_), 4.08, 3.97 (each 1H, dd, *J*_A_ = *J*_B_ = 9.0 Hz, 20-CH_2_), 3.30 (1H, d, *J* = 9.0 Hz, 13-CH), 2.79 (2H, m, 30-CH_2_), 2.62 (2H, m, 31-CH_2_), 2.42 (2H, m, 12-CH_2_), 2.03 (1H, m, 5-CH), 1.97 (1H, m, 9-CH), 1.70~1.81 (4H, m, 3,11-CH_2_), 1.24(2H, m, 2-CH_2_), 1.03 (3H, s, 18-CH_3_), 0.96 (3H, s, 19-CH_3_); MS (ESI) *m*/*z*: 829.4 [M + Na]^+^; HR-MS (ESI, M + NH_4_) *m*/*z*: calcd for C_40_H_46_N_3_O_14_S: 824.2695, found: 824.2697.

### 3.2. Biology

#### 3.2.1. Antibacterial Assay

The minimal inhibitory concentrations (MICs) were carried out by microbroth dilution method described by the Clinical Laboratory Standards Institute [30,31]. A stock solution of all the target compounds was prepared with DMSO-medium (1:2), and then graded quantities of the test compounds were diluted with medium. The specified concentration suspension of fungus and bacterium contained approximately 10^3^, and 10^5^ CFU/mL was added into microtitration plates. The inoculated 96-well plates were incubated at 35 °C for 18 h. MIC was defined as the lowest concentrations that prevented visible growth of the bacteria.

#### 3.2.2. MTT Assay

MTT assay was developed to monitor mammalian cell survival and proliferation *in vitro* [32]. CaEs-17 cells (K562, Bel-7402 and MGC-803 cells) from American Type Culture Collection (ATCC, Manassas, VA, USA) were cultivated in RPMI-1640 medium supplemented with 5% fetal bovine serum (*v*/*v*), 100 U/mL penicillin, and 50 mg/mL streptomycin. Cells (5 × 10^4^ cell/mL) at the log phase of their growth cycle were added to each well of a 96-well plate (100 µL/well) and incubated for 24 h at 37 °C in a humidified atmosphere of 5% CO_2_. Then, cells were treated with or without test compounds in different concentrations. After 72 h, 5 mg/mL MTT solution (20 µL per well) was added. Cells were incubated at 37 °C. After 4 h, MTT solution was removed and DMSO was added to each well (150 µL). Ten minutes later at room temperature, the optical density (OD) values were measured at the wavelength of 490 nm on a Microplate Reader NO. 550 (BIO-RAD Instruments Inc., Hercules, CA, USA). In this experiment, 10 µg/mL of Taxol was used as the positive control and 0.1% DMSO was used as the negative reference.

#### 3.2.3. Griess Assay

NO produced by the compounds was determined by assaying the levels of NO_2_ using the Griess reagent [33]. The levels of NO released from 100 μM of each compound were tested by nitrate/nitrite in triplicate. The lysates were reacted with Griess reagent (40 min), centrifugalized (10 min), and then measured at 540 nm. This assay was carried out according to the manufacturer’s instructions for 60 min (Beyotime, Nanjing, China).

#### 3.2.4. Cell Cycle Arrest

Bel-7402 cells (5.0 × 10^3^ cells/well) were plated in 6-well plates and incubated for 24 h, and then incubated with different concentrations (0.25, 0.5 and 1 μM) of **9f** at 37 °C for 48 h. After 48 h treatment, cells were treated with 70% ethanol and RNase, sequentially, and stained with PI [34]. Cellular DNA content was measured by a flow cytometer for cell cycle distribution analysis.

#### 3.2.5. Cellular Apoptosis

Bel-7402 cells were incubated with different concentrations (0.25, 0.5 and 1 μM) of **9f** for 72 h. Then, Annexin-V and PI double staining was used to detect apoptotic cells to analyze apoptosis according to the manufacturer’s instructions by flow cytometry [35].

#### 3.2.6. Mitochondrial Membrane Potential

Bel-7402 cells were cultured in six-well plates after treatment with **9f** or vehicles for 48 h. The cells were cells were collected by trypsinization, washed with PBS (Sigma Chemical Co., Shanghai, China) and stained according to the manufacturer’s instruction (Keygen, KGA601, Nanjing, China) with the lipophilic cationic dye JC-1. The flow cytometry analysis was carried out to monitor the percentage of cells with healthy or collapsed mitochondrial membrane potentials [36].

## 4. Conclusions

In summary, a series of NO donor/enmein-type diterpenoid derivatives (**9a**–**i**) were synthesized from relevant commercial available resources. All of the target compounds showed antibacterial activity against Gram-positive bacteria *S. aureus* and *B. subtilis* and antiproliferative activity against the K562 leukemia cell line, the MGC-803 gastric cancer cell line, the CaEs-17 esophageal cancer cell line, the Bel-7402 hepatoma cell line, and human normal liver cells L-02 to some extent. It was found that **9b** and **9d** with the same total linkage length of five carbons were the most active against *S. aureus* and *B. subtilis* with MICs of 4 and 2 μg/mL, respectively. Compound **9f** displayed IC_50_ values of 1.68, 1.11, 3.60 and 0.72 μM against four tested cancer cell lines, respectively, and 18.80 μM against normal liver cells L-02. The SI value was 26.1 between L-02 normal liver and Bel-7402 hepatoma cell lines. The preliminary SARs were also concluded based on the obtained biological evaluation data. Furthermore, it was also found that **9f** induced apoptosis via the mitochondria-related pathway and arrested cell cycle of Bel-7402 cells at the S stage. These findings were important to explore new chemical entities with innovative natural scaffolds for the treatment of infection combined with solid tumors.

## Figures and Tables

**Table 1:** The antimicrobial activity of the synthesized compounds (MIC μg/mL).

Compound	*E. coli*	*S. aureus*	*B. subtilis*	*M. albicans*
**7**	>100	32	32	>100
**8**	>100	>100	>100	>100
**9a**	>100	16	64	>100
**9b**	>100	4	2	>100
**9c**	>100	8	4	>100
**9d**	>100	4	2	>100
**9e**	>100	16	16	>100
**9f**	>100	32	64	>100
**9g**	>100	16	16	>100
**9h**	>100	16	16	>100
**9i**	>100	16	2	>100
Chloromycetin	4	4	8	NT ^1^
Fluconazole	NT	NT	NT	4

^1^ NT, not test.

**Table 2:** Antiproliferative activity of the synthesized compounds (IC_50_ μM).

Compound	K562	MGC-803	CaEs-17	Bel-7402	L-02	SI ^2^
**7**	4.76 ± 0.32	5.69 ± 0.39	11.03 ± 1.02	7.48 ± 0.53	18.26 ± 0.81	2.4
**8**	8.11 ± 0.76	14.21 ± 1.22	30.84 ± 2.09	32.96 ± 2.19	24.37 ± 1.59	0.7
**9a**	2.47 ± 0.16	1.83 ± 0.16	5.12 ± 0.42	1.33 ± 0.10	10.36 ± 0.61	7.7
**9b**	2.31 ± 0.21	1.62 ± 0.08	4.83 ± 0.30	1.20 ± 0.08	16.73 ± 0.17	13.9
**9c**	1.93 ± 0.10	1.34 ± 0.13	3.76 ± 0.37	0.83 ± 0.06	19.87 ± 0.18	23.9
**9d**	2.15 ± 0.12	1.50 ± 0.10	4.98 ± 0.42	1.23 ± 0.08	13.58 ± 1.31	11.0
**9e**	1.92 ± 0.09	1.29 ± 0.12	4.27 ± 0.35	0.92 ± 0.05	17.20 ± 0.77	18.6
**9f**	1.68 ± 0.12	1.11 ± 0.05	3.60 ± 0.12	0.72 ± 0.04	18.80 ± 1.25	26.1
**9g**	2.26 ± 0.08	1.43 ± 0.08	5.13 ± 0.22	1.27 ± 0.11	11.57 ± 0.39	9.1
**9h**	2.11 ± 0.16	1.49 ± 0.11	4.68 ± 0.31	1.08 ± 0.05	12.09 ± 1.08	11.1
**9i**	1.86 ± 0.18	1.25 ± 0.10	3.82 ± 0.19	0.78 ± 0.04	14.57 ± 0.86	18.6
**Taxol ^1^**	0.41 ± 0.02	0.85 ± 0.06	0.43 ± 0.03	1.89 ± 0.09	3.73 ± 0.17	1.9

^1^ Taxol was used a positive control. Data were means ± SD of three experiments; ^2^ SI: selectivity index. It was calculated as: SI = IC_50, L-02_/IC_50, Bel-7402_.

## Supplementary Materials

Supplementary materials can be found at http://www.mdpi.com/1422-0067/17/6/747/s1, including ^1^H NMR and H-H COSY of 8, ^1^H and ^13^C NMR of methyl ester of **6a**, and ^1^H and ^13^C NMR of **9f**.

## Abbreviations

NO	Nitric Oxide
SAR	Structure Activity Relationship
DMAP	4-Dimethylaminopyridine
EDCI	1-Ethyl-3-(3-dimethylaminopropyl)carbodiimide Hydrochloride
rt	Room Temperature
THF	Tetrahydrofuran
SI	Selectivity Index
MTT	3-(4,5-Dimethylthiazol-2-yl)-2,5-diphenyltetrazolium bromide
TLC	Thin Layer Chromatograph
FBS	Fetal Bovine Serum
TMS	Tetramethylsilane
^1^H NMR	Proton Nuclear Magnetic Resonance
TMS	Tetramethlysilane
ESI MS	Electrospray Ionization-Mass Spectrometry
HR-MS	High Resolution Mass Spectrum
DCM	Dichloromethane
MIC	Minimal Inhibitory Concentration
PI	Propidium Iodide
NT	Not Test
