# Supplementary material for: Synthesis, Biological Activity, and Apoptotic Properties of NO-Donor/Enmein-Type ent-Kauranoid Hybrids
Source: Int J Mol Sci. 2016 May 24;17(6):747. doi: 10.3390/ijms17060747 (PMC4926326; doi:10.3390/ijms17060747)
Supplement: Supplementary file 1 [file ijms-17-00747-s001.pdf]

Supplementary Materials: Synthesis, Biological Activity, and Apoptotic Property of NO-Donor/Enmein-Type *ent*-Kauranoid Hybrids

Dahong Li, Xu Hu, Tong Han, Shengtao Xu, Tingting Zhou, Zhenzhong Wang, Keguang Cheng, Zhanlin Li, Huiming Hua, Wei Xiao and Jinyi Xu

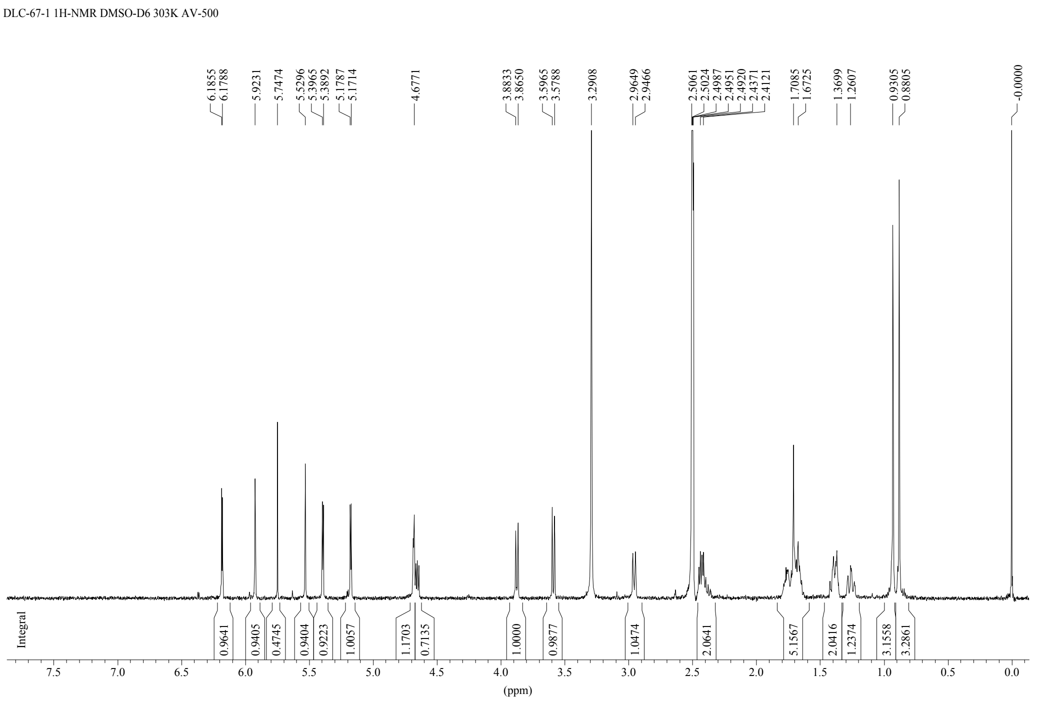

Figure S1. ^{1}H-NMR spectrum of compound **8** (500 MHz, DMSO-d_6).

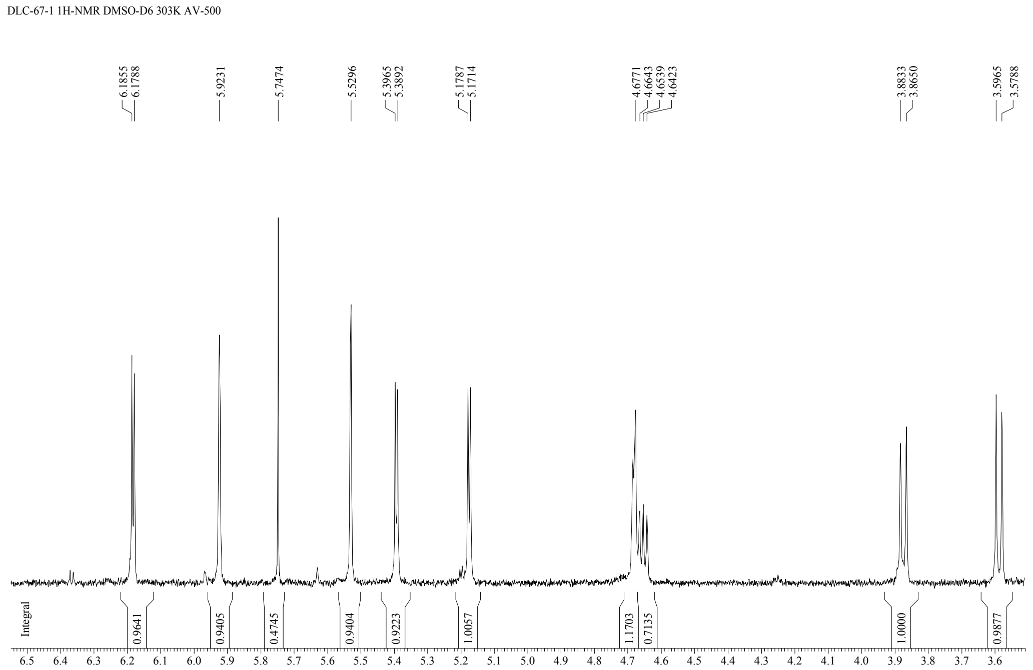

Figure S2. Region amplified ^{1}H-NMR spectrum 2-1 of **8** (500 MHz, DMSO-d_6).

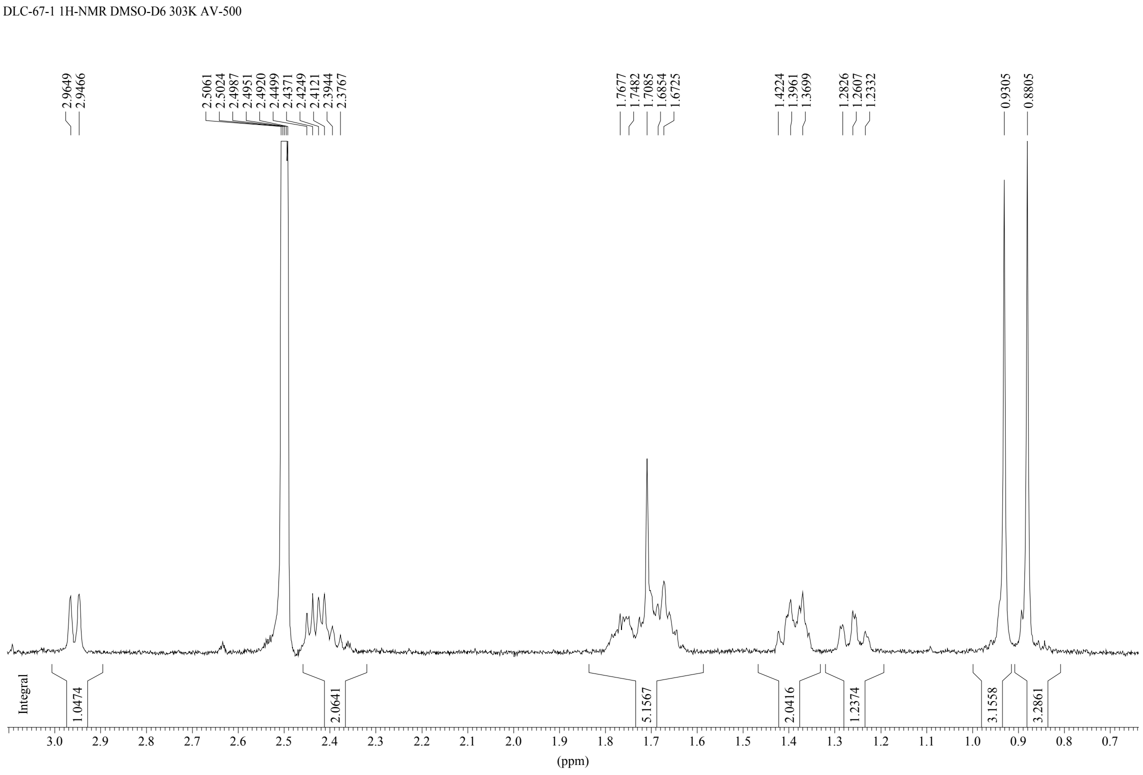

Figure S3. Region amplified ^{1}H-NMR spectrum 2-2 of **8** (500 MHz, DMSO-d_6).

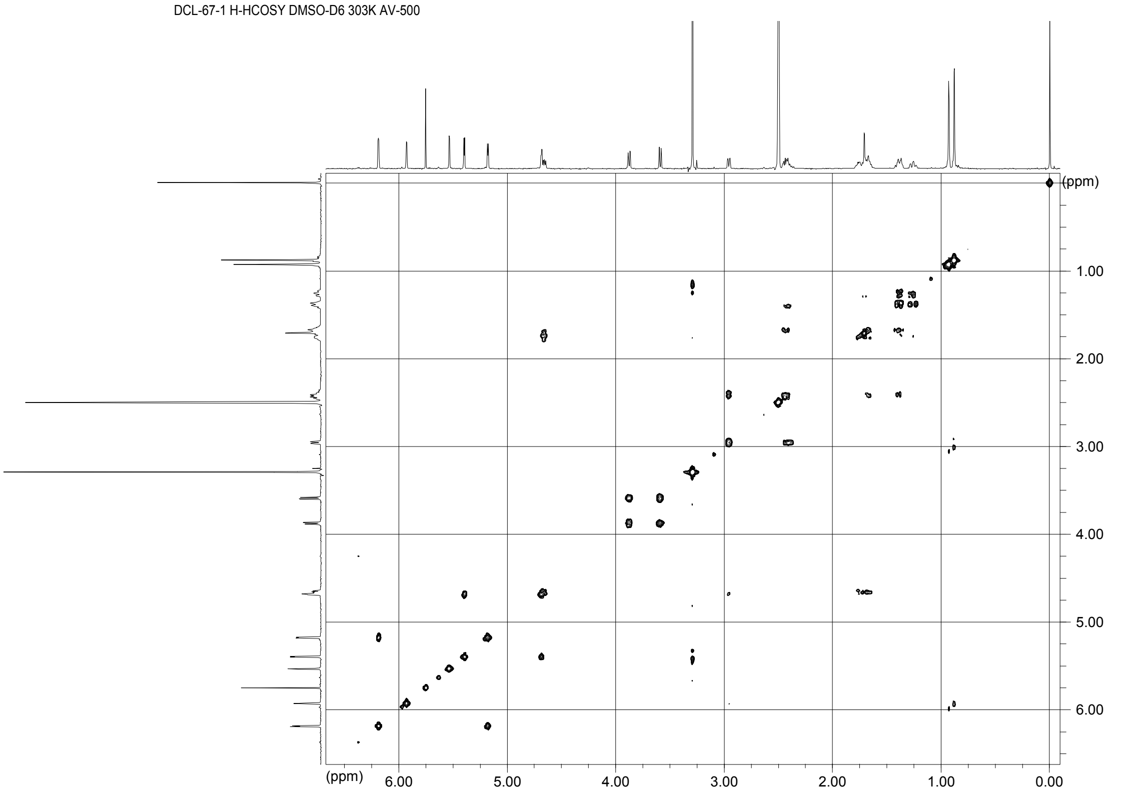

Figure S4. H-H COSY spectrum of **8** (500 MHz, DMSO-d_6).

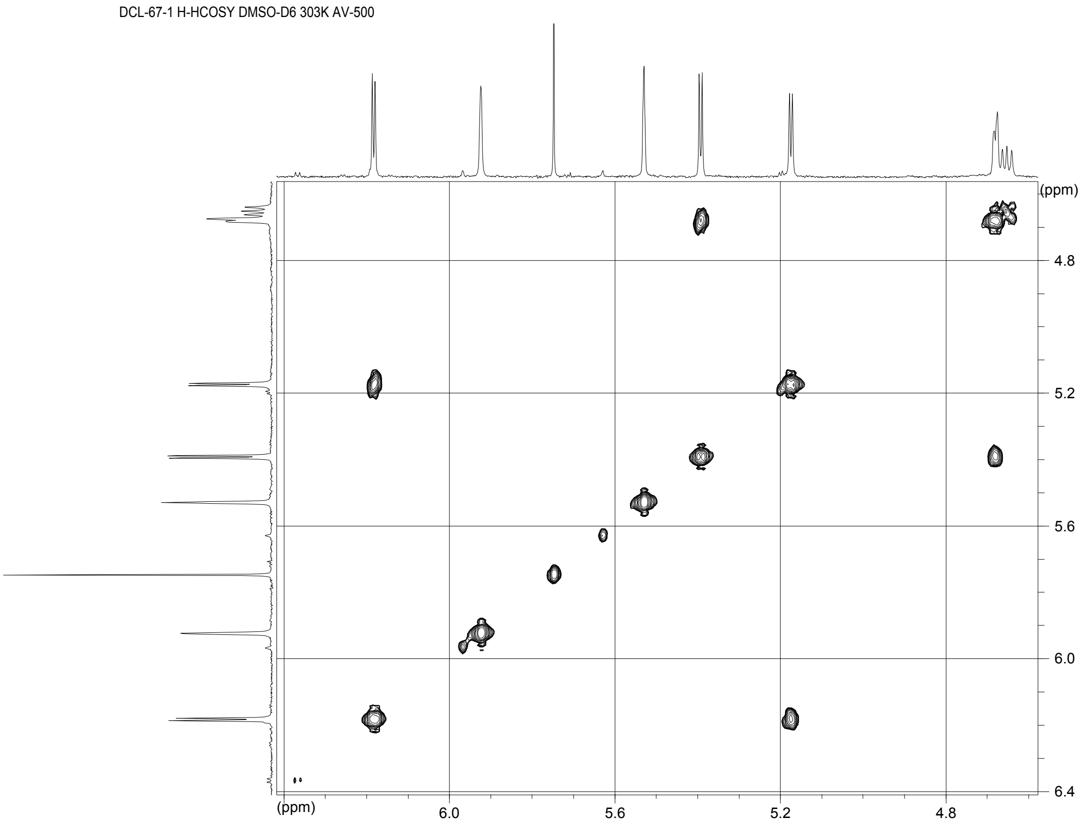

Figure S5. Region amplified H-H COSY spectrum 3-1 of **8** (500 MHz, DMSO-d_6).

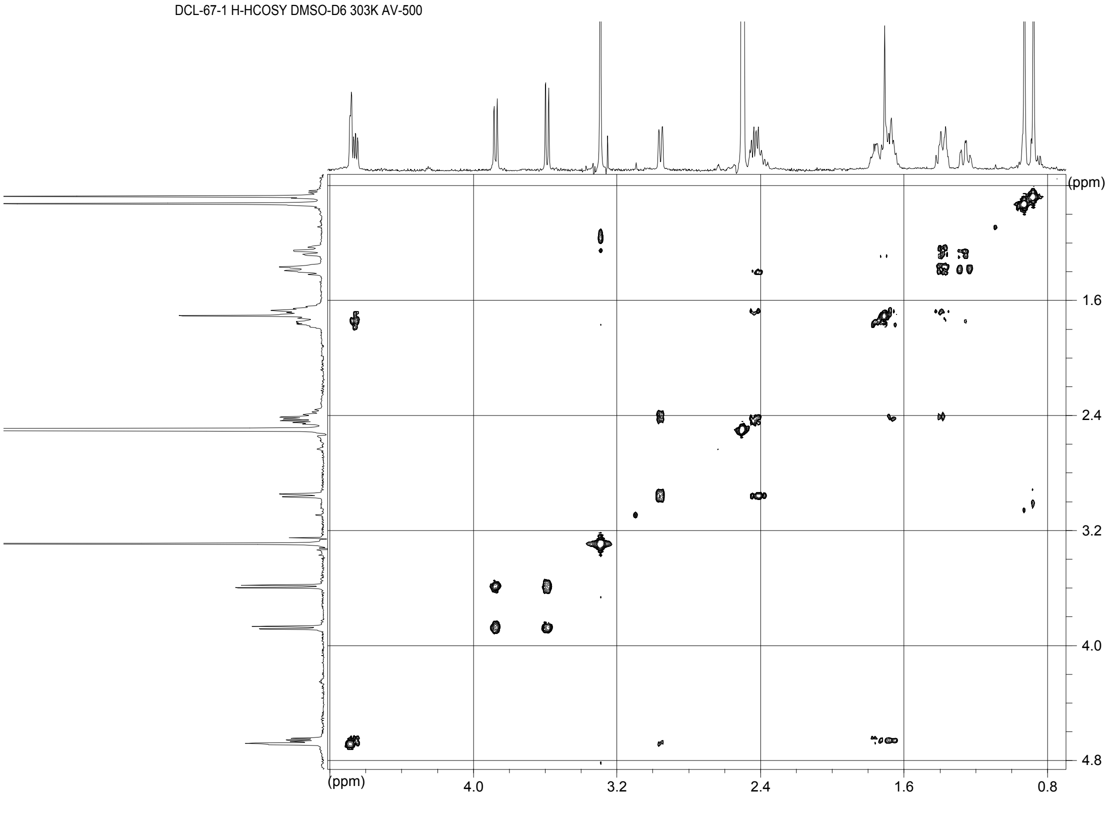

Figure S6. Region amplified H-H COSY spectrum 3-2 of **8** (500 MHz, DMSO-d_6).

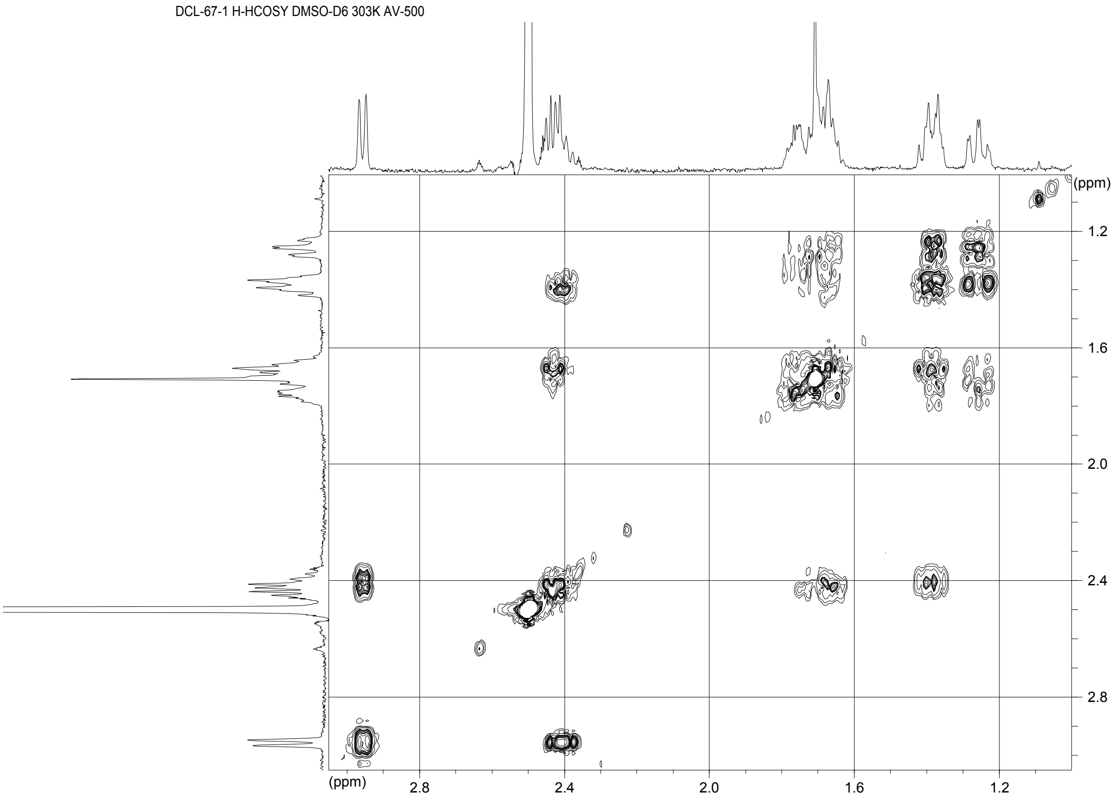

Figure S7. Region amplified H-H COSY spectrum 3-3 of **8** (500 MHz, DMSO-d_6).

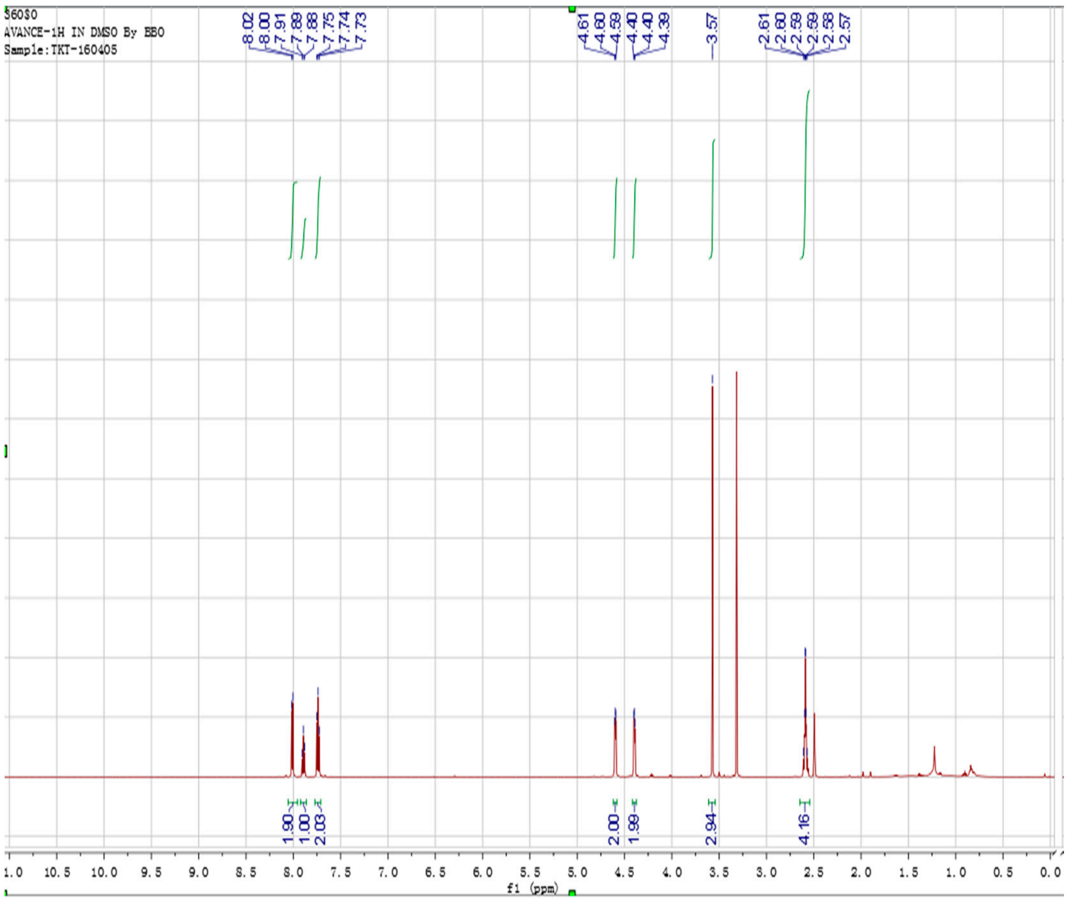

Figure S8. ^{1}H-NMR spectrum of the methyl ester of **6a** (400 MHz, $CDCl_3$).

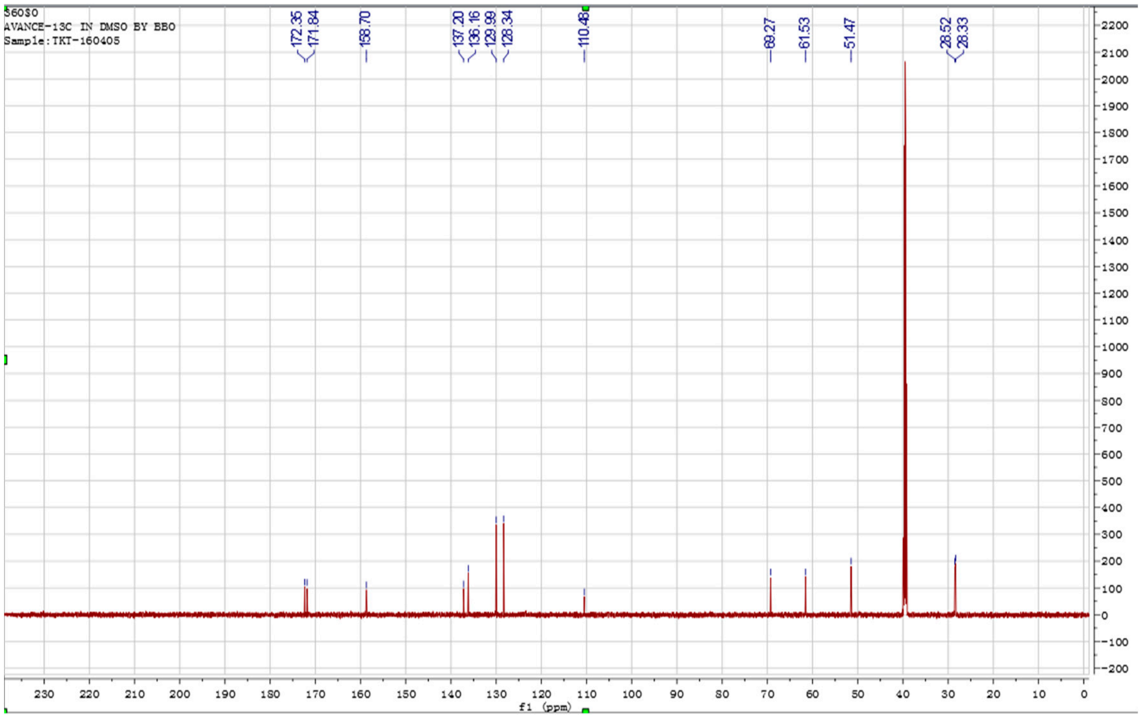

Figure S9. ^{13}C-NMR spectrum of the methyl ester of **6a** (100 MHz, $CDCl_3$).

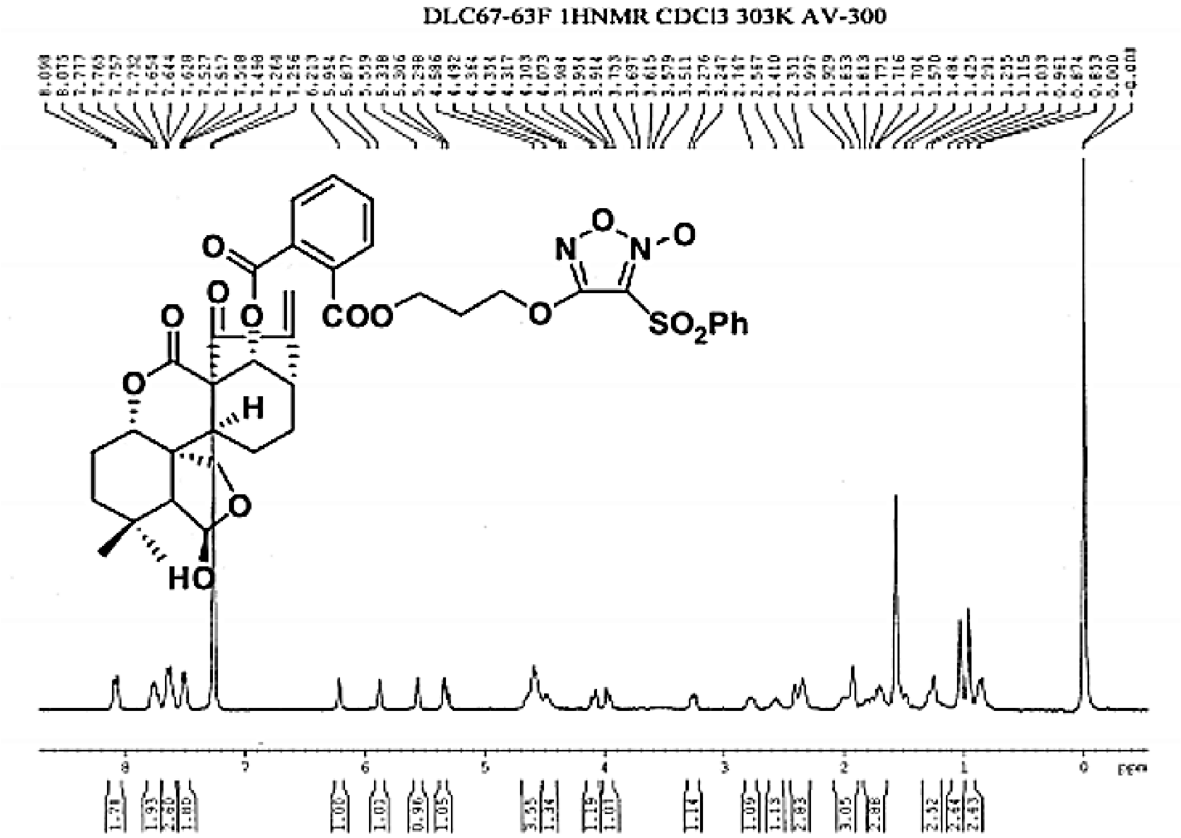

Figure S10. ^{1}H-NMR spectrum of **9f** (300 MHz, $CDCl_3$).

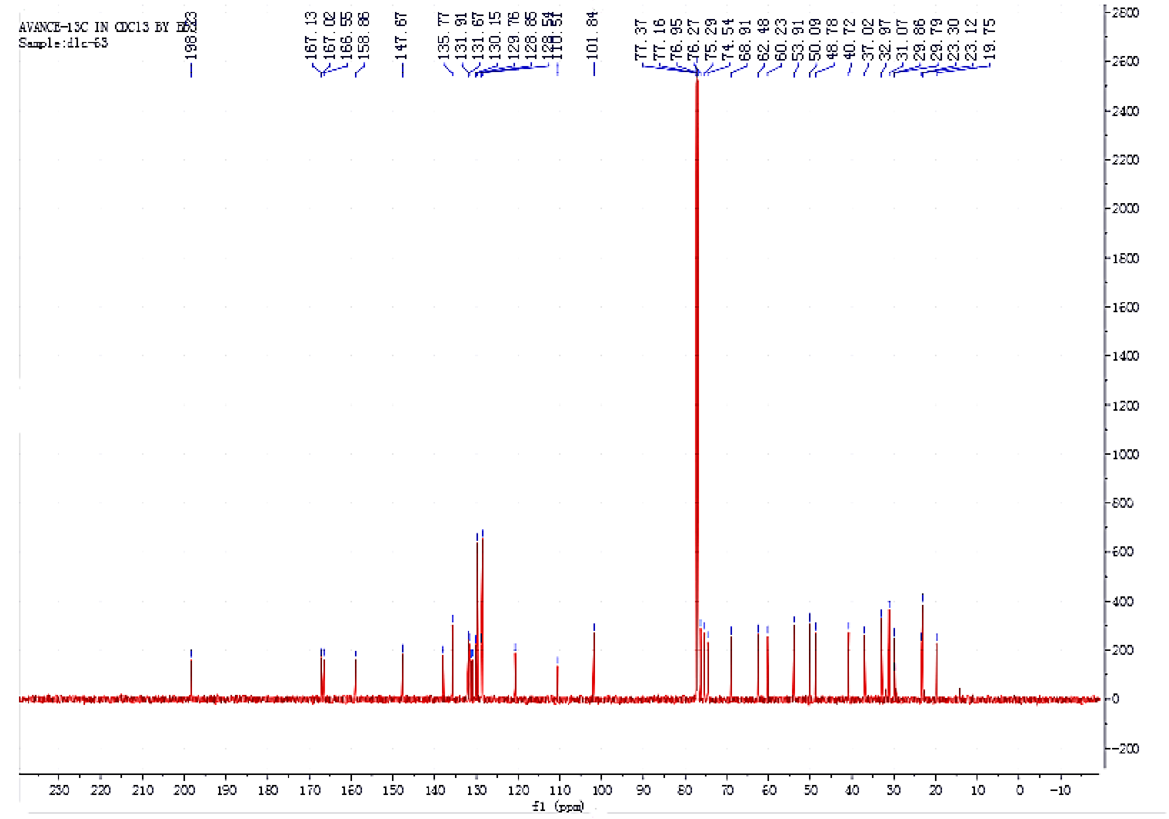

Figure S11. ^{13}C-NMR spectrum of **9f** (100 MHz, $CDCl_3$).

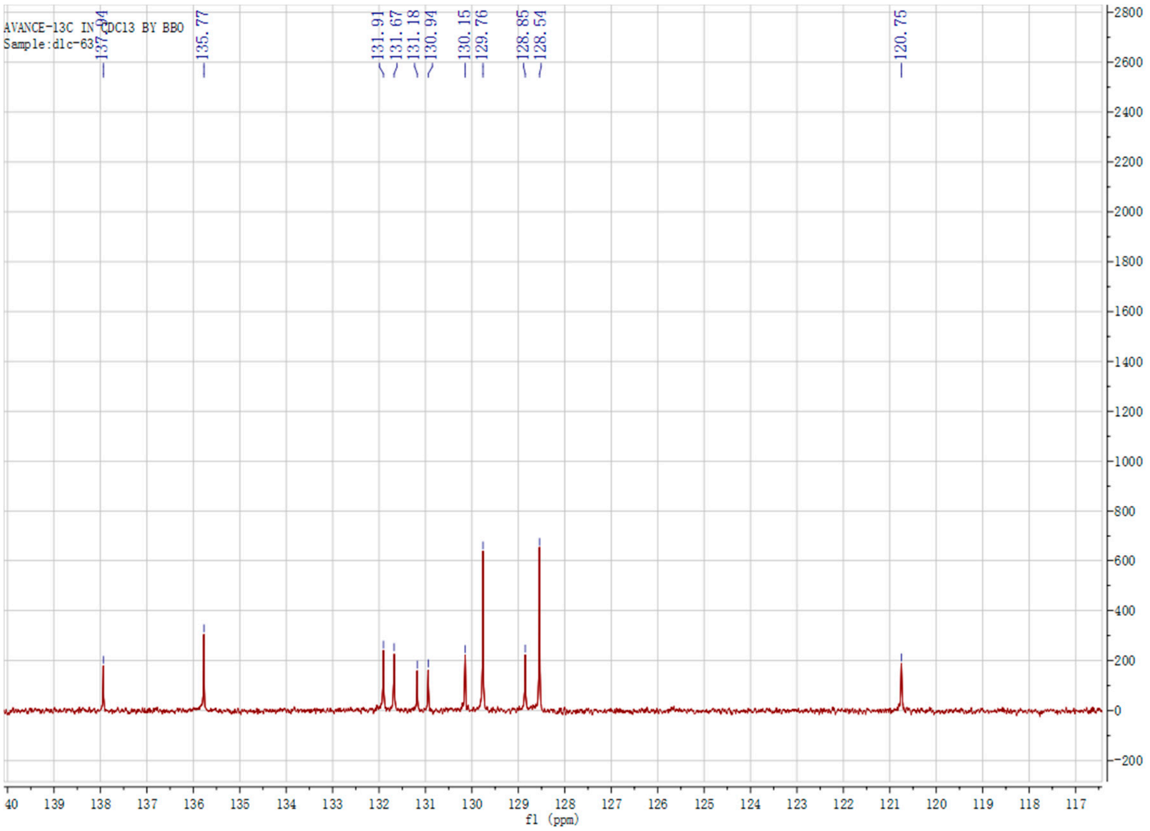

Figure S12. Region amplified ^{13}C-NMR spectrum of **9f** (100 MHz, $CDCl_3$).
